# Angiolymphoid hyperplasia with eosinophilia developing in a patient with history of peripheral T-cell lymphoma: evidence for multicentric T-cell lymphoproliferative process

**DOI:** 10.1186/1746-1596-3-22

**Published:** 2008-05-29

**Authors:** Luis F Gonzalez-Cuyar, Fabio Tavora, X Frank Zhao, Guanghua Wang, Aaron Auerbach, Nadine Aguilera, Allen P Burke

**Affiliations:** 1 Department of Pathology, University of Maryland School of Medicine, Baltimore, Maryland, USA; 2 Department of Hematopathology, Armed Forces Institute of Pathology, Washington DC, USA; 3 Department of Molecular Diagnostics, Armed Forces Institute of Pathology, Washington DC, USA

## Abstract

**Background:**

Angiolymphoid hyperplasia with eosinophilia (ALHE) is a vasocentric process characterized by infiltrates of lymphocytes and eosinophils, usually affecting the muscular arteries of the head and neck. Currently it is unclear whether it is a reactive or neoplastic process.

**Report:**

We present a 61-year-old African American male with a twenty year history of superficial skin patches involving the head and neck region. An excisional biopsy of a right submental lymph node revealed an atypical T-cell lymphocytic process, diagnosed as peripheral T-cell lymphoma after immunophenotyping and molecular studies. Three months later the patient underwent a biopsy of a left temporal nodule that was diagnosed as ALHE. Subsequently, at two year follow-up, the patient was diagnosed with Mycosis Fungoides. Polymerase chain reaction for T cell receptor gamma showed the same T-cell receptor gene rearrangement in both the temporal mass and the right submental lymph node.

**Conclusion:**

ALHE with molecular evidence of monoclonality is extremely unusual, as is the association with nodal peripheral T-cell nodal lymphoma. The findings of this case support our hypothesis that ALHE might be an early form of T-cell lymphoma.

## Introduction

ALHE is characterized clinically by single to multiple red brown dome shaped papules or subcutaneous nodules located mainly in the head and neck [1-4]. In some cases the nodules extend to the dermis or into the muscle. About 1/5 of patients have blood eosinophilia and lymphadenopathy[2]. Histologically the lesions are characterized by a reactive proliferation of small blood vessels, often surrounding a muscular artery, with peripheral inflammatory infiltrates consisting of mononuclear cells and eosinophils. The reactive blood vessels are often epithelioid, leading to the terms "histiocytoid" or, more recently "epithelioid" hemangioma[5]. Immunohistochemical stains usually show a major population of T lymphocytes[6] with occasional B cells forming lymphoid follicles[5]. Since the description of the initial large series [5], there have been numerous reports of this condition, with lesions occurring in a variety of organs, including disseminated disease[1,7-13].

The etiology of ALHE is unknown. It is not clear if it is primarily a vascular neoplasm, as suggested by an alternate name (epithelioid hemangioma), a lymphoproliferative process, or a heterogeneous group of entities. There is some evidence that it may be related to traumatic pseudoaneurysm, supporting a vascular origin[14]. More recent data suggest that ALHE may be a primary lymphoproliferative process, as evidenced by findings of T-cell gene rearrangements, although PCR analysis has not shown monoclonality in all cases[15]. There has been a single report of a patient with ALHE who subsequently developed peripheral T-cell lymphoma [16]

The purpose of our study is to report the first documented case of ALHE developing after the diagnosis of peripheral T-cell lymphoma with T-cell receptor gene rearrangements showing monoclonality in both the lymphoma and the vascular lesion.

## Case presentation

We present the case of a 61-years-old African American male patient with history of hypertension and asthma. The patient had a 20–30 year history of superficial skin patches over the torso, and neck that over the five to six years prior to the current presentation progressed to a diffusely pruritic maculopapular rash with multiple subcutaneous skin nodules involving the head and neck region. The patient reported that over in that period of time multiple biopsies of the nodules yielded nonspecific diagnoses and was treated with doxycycline and dapsone steroids. The largest nodule measured 3.5 × 2.5 cm and was located on the left temporal scalp. A second nodule on the right forehead measured 3.5 × 2.0 cm, multiple smaller nodules were also noted.

**Figure 1:**
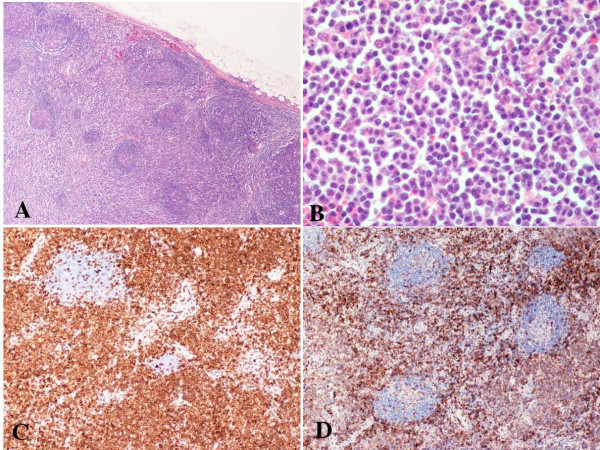
Peripheral T-cell lymphoma, unspecified (submental lymph node biopsy). A, The H&E section demonstrates expansion of the interfollicular T-cells (low magnification); B. The infiltrating T-cells show atypia and clear cytoplasm (high magnification); C, Paraffin immunoperoxidase staining reveals the lymphoma cells are positive for CD4; D. Reactive CD8-positive T-cells are also present.

The patient underwent a fine needle aspiration (FNA) of a right submental nodule that revealed a T-cell lymphoproliferative disorder. An excisional biopsy was performed which revealed a largely effaced lymph node with small follicular centers, and marked paracortical expansion in a background of macrophages and eosinophils (Figure 1). Immunohistochemical markers show atypical cortical lymphocytes that were positive for CD3, CD5, and CD43 and negative for CD7, CD15, CD20 and CD30. The small follicular centers wee positive for CD20 and CD23. Flow cytometry revealed CD45 dim lymphocytes expressing CD2, CD3, CD4, CD5, and CD20 and were negative for CD8, CD10, CD11c, CD16, CD19, CD25, CD23, CD38, CD56, CD57, kappa and lambda. It was diagnosed as suspicious for T-cell lymphoma. CT scans demonstrated axillary, inguinal and borderline hilar lymphadenopathy. A complete blood count CBC revealed eosinophilia at 19%.

**Figure 2:**
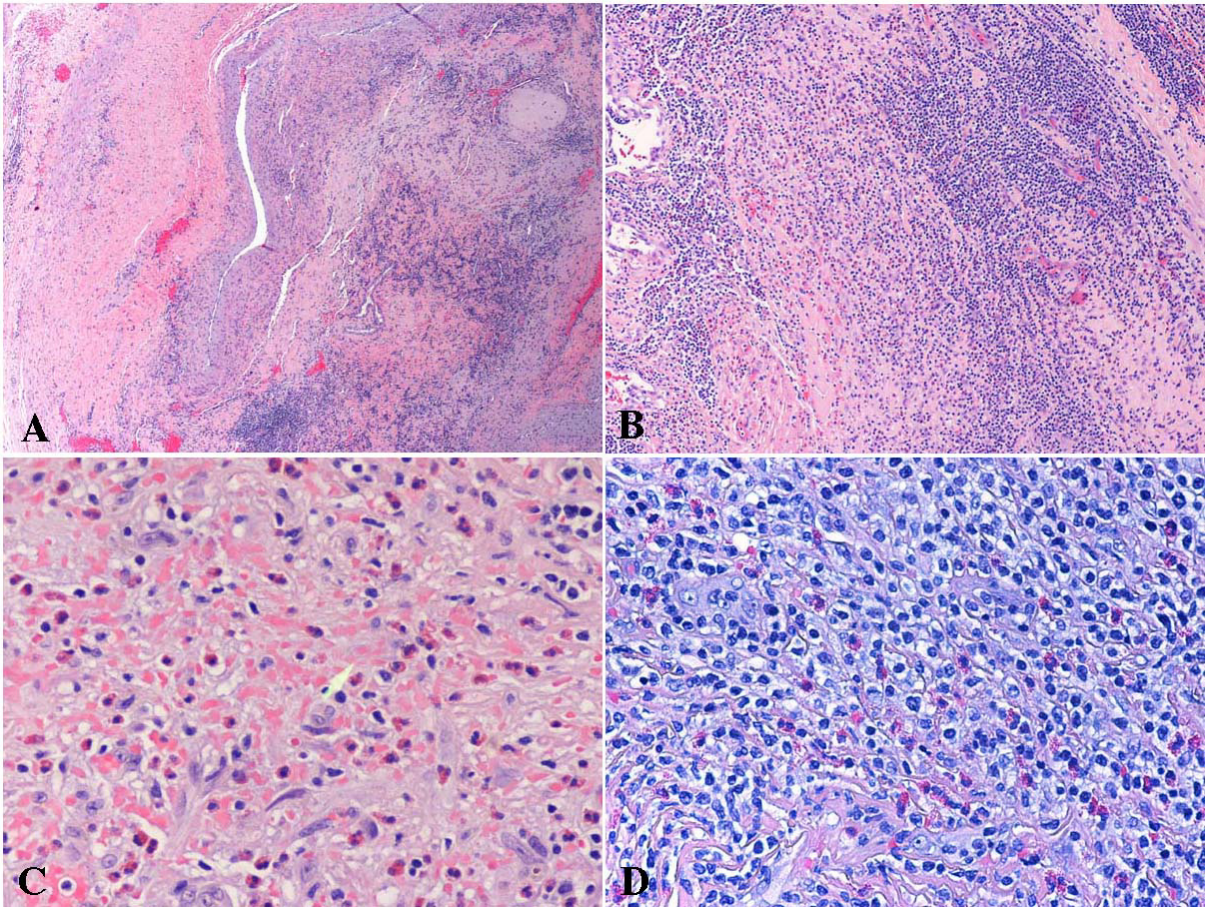
Angiolymphoid hyperplasia with eosinophilia (vessel, temporal region, biopsy). A. Low magnification demonstrates a vessel wall infiltrated by small lymphoid cells. B. Higher magnification demonstrates a population of lymphoid cells with prominent vascularity. C. There are focally increased eosinophils and reactive "epithelioid" endothelial cells. D. The atypical lymphoid cells show small amount of clear cytoplasm with scattered eosinophils in the background.

Three months later a biopsy of the left temporal nodule revealed a muscular artery with medial disruption and thrombosis, chronic inflammation and eosinophils. The surrounding vascular proliferation had thick walls and was notable for plump endothelial cells with hyperchromatic nuclei (Figure 2). The diagnosis of ALHE was rendered. Ten months after the initial presentation the patient presented with progressive lymphadenopathy. At this time peripheral eosinophilia was also noted at 15.6%.

At twenty-two months follow up the patient was diagnosed with mycosis fungoides and began pentostatin chemotherapy. The patient received six cycles with significant resolution of his pruritus and decrease in size of the nodules. Subsequent CT scans demonstrated decreased lymphadenopathy.

Tissue from the right submental nodule (T-cell lymphoma) as well as from the left temporal nodule (ALHE) were analyzed by T cell receptor gene rearrangement studies and revealed identical monoclonal bands in TCR gamma assay. In our internal validation studies, a monoclonal band was detected in 80% of T-cell lymphoma and 8.7% of B cell lymphoma in TCR gamma assay. Biclonal monoclonal bands are not commonly seen in leukemia/lymphoma cases. It occurs in less than 10% of leukemia/lymphoma cases with clonality. There is no dominant monoclonal band observed in IgH and TCR beta assays for both specimen A and B.

## Discussion

Angiolymphoid hyperplasia with eosinophilia (ALHE) is a rare condition affecting muscular arteries, typically of the head and neck[1]. It was first described in 1969 by Wells and Whimster[4]. They reported nine patients between the ages of 19 and 43, five women and four men, with single to multiple lesions in the head and neck region with blood eosinophillia in all patients and regional lymphadenopathy in four of nine patients[4]. Previously it had been described as pseudo- or atypical pyogenic granuloma, subcutaneous angioblastic lymphoid hyperplasia with eosinophilia, and papular angioplasia[1,5]. Initially thought to be related to Kimura's disease, a condition occurring in male Asians sharing some of the same clinical and histological features, ALHE is now considered a distinct entity[2,5,17,18].

In approximately 50% of cases, a muscular artery is at the center of the lesion, as in our case report. Occasionally, in such cases, the differential diagnosis is traumatic pseudoaneurysm, [19] although in the latter condition, the lymphoid infiltrate and eosinophilic response is generally minimal.

Although the lymphoid infiltrate is a prominent component of ALHE, there are few data supporting a primarily lymphoproliferative process for this condition. In a study conducted by Jang et al[15], two of seven cases of ALHE showed positive result for PCR analysis of rearranged TCR-gene; however, all the cases were negative for heteroduplex-PCR[15]. The conclusion of these authors was that the lymphoid reaction in ALHE is most likely reactive. However, more recently, Kempf et al [2] demonstrated T-cell gene rearrangements in 5 of 7 cases of ALHE and monoclonality was confirmed by automated high-resolution PCR fragment analysis. These authors raised the question of whether ALHE or a subset of ALHE represents either a true T-cell lymphoma of low-grade malignancy or a specific variant of reactive lymphoid hyperplasia [2]. In our current case PCR analysis of the TCR-gene showed monoclonality between the peripheral T-cell lymphoma and the ALHE specimens.

Further support that ALHE is a monoclonal T-cell process is the finding of ALHE confirmed histologically in patients with synchronous or metachronous T-cell lymphoma. Adreae et al [16] reported a young girl with ALHE who subsequently developed peripheral T-cell lymphoma years after initial diagnosis. The current report demonstrates a second case, in which the ALHE developed months after the diagnosis of peripheral T-cell lymphoma. The finding of ALHE and peripheral T-cell lymphoma, and the demonstration of T cell gene rearrangements in both tissues, has not been previously documented in a single patient.

Although the current patient supports the concept that ALHE is, at least in some patients, reflected of a T-cell lymphoproliferative process, the finding of T-cell gene rearrangements indicated of monoclonality. According to Kempf et al [2], clonal lesional lymphocytes cannot be considered synonymous with malignant potency or overt malignancy, but it does shed a new light on the pathogenetic aspects of this disorder.

In conclusion, we report a case of ALHE developing after the diagnosis of peripheral T-cell lymphoma with T-cell gene rearrangements studies showing monoclonality in both the lymphoma and vascular lesions.

## Authors' contributions

FT, XFZ, GW, APB carried out the molecular genetic studies, participated in the sequence alignment and drafted the manuscript, LFGC, XFZ, APB carried out the immunoassays, MT participated in the sequence alignment, AA, NA, GW participated in the design of the study and performed the statistical analysis, LFGC, FT conceived of the study, and participated in its design and coordination. All authors read and approved the final manuscript.
